# Splenectomy Prior to Experimental Induction of Autoimmune Hepatitis Promotes More Severe Hepatic Inflammation, Production of IL-17 and Apoptosis

**DOI:** 10.3390/biomedicines9010058

**Published:** 2021-01-09

**Authors:** Laura Elisa Buitrago-Molina, Janine Dywicki, Fatih Noyan, Martin Trippler, Julia Pietrek, Jerome Schlue, Michael P. Manns, Heiner Wedemeyer, Elmar Jaeckel, Matthias Hardtke-Wolenski

**Affiliations:** 1 Department of Gastroenterology, Hepatology & Endocrinology, Hannover Medical School, D-30625 Hannover, Germany; Buitrago.Laura@mh-hannover.de (L.E.B.-M.); janine.dywicki@gmx.de (J.D.); noyan.fatih@mh-hannover.de (F.N.); Manns.Michael@mh-hannover.de (M.P.M.); Wedemeyer.Heiner@mh-hannover.de (H.W.); Jaeckel.Elmar@mh-hannover.de (E.J.); 2 Department of Gastroenterology and Hepatology, University Hospital Essen, University Duisburg-Essen, D-45147 Essen, Germany; Martin.Trippler@uk-essen.de (M.T.); Julia.Pietrek@uk-essen.de (J.P.); 3 Institute of Pathology, Hannover Medical School, D-30625 Hannover, Germany; schlue.jerome@mh-hannover.de

**Keywords:** autoimmune hepatitis, experimental murine hepatitis, liver, spleen, IL-17, apoptosis, inflammation, caspase-3, adenovirus, formiminotransferase cyclodeaminase

## Abstract

Autoimmune hepatitis (AIH) is detected at a late stage in the course of the disease. Therefore, induction and etiology are largely unclear. It is controversial if the induction of autoimmunity occurs in the liver or in the spleen. In our experimental murine AIH model, the induction of autoimmunity did not occur in the spleen. Instead, a protective role of the spleen could be more likely. Therefore, we splenectomized mice followed by induction of experimental murine AIH. Splenectomized mice presented more severe portal inflammation. Furthermore, these mice had more IL-17, IL-23 receptor (IL-23R) and caspase 3 (casp3) and a decreased amount of erythropoietin in serum, while intrahepatic T cell compartments were unaffected. These results indicate that the spleen is not necessary for induction of AIH, and splenectomy disrupts the ability to immune regulate the intensity of hepatic inflammation, production of IL-17 and apoptosis.

## 1. Introduction

Autoimmune hepatitis (AIH) is a chronic autoimmune inflammation directed against liver tissue. Patients are usually identified late in the course of the disease, due to the development of AIH being associated with a long delay between the onset of autoimmunity and the diagnosis of the symptomatic disease [1,2]. Due to the delayed onset of the disease, it is still difficult to identify the important factors and to prevent the induction of the disease. In addition, little is known about the etiology of AIH, as there are few reliable animal models that reflect the disease [3,4,5,6,7,8]. One of the controversies revolves around, among other things, the place of origin of both human and murine AIH. Some groups have shown that the spleen is essential for triggering an immune response to hepatocytes [9,10], while we and others have shown that the liver is important for the induction [11,12,13]. Rather, we speculated that the spleen has a protective function in our experimental murine AIH (emAIH) model.

Therefore, we analyzed the induction phase more in detail and were able to show that the absence of the spleen promoted the immunopathologic course of AIH. We identified IL-17, IL-23R and casp3 as the main factors associated with hepatic portal inflammation. Furthermore, this effect was exacerbated by a lack of erythropoietin (Epo).

## 2. Materials and Methods

### 2.1. Ethics Statement

Animal care and experiments were performed in accordance with institutional and national guidelines. All animal experiments were performed according to protocols approved by the animal welfare commission of the Hannover Medical School and local ethics animal review board (Lower Saxony State Office for Consumer Protection and Food Safety, Oldenburg, Germany).

### 2.2. Mice

Male NOD/Ltj mice were bred and maintained under specific pathogen-free conditions at the central animal facility of the Hannover Medical School (Hannover, Germany). Mice were injected intravenously with a total of 4 × 10^9^ infectious particles in PBS of Ad-FTCD. Some mice were splenectomized two weeks before administration of adenovirus, expressing formiminotransferase cyclodeaminase (Ad-FTCD). All mice were sacrificed 12 weeks post infection.

### 2.3. Adenovirus Construction

The generation of Ad-FTCD was described before [3]. In brief, FTCD was amplified by PCR from cDNA generated from human liver cells; its sequence was verified by the sequencing of both DNA strands. The constructs were fused to the Ad transfer vector pShuttle-CMV (Stratagene, Waldbronn, Germany). By homologous recombination, this shuttle vector was recombined with pAdEasy-1, which carries deletions in the E1- and E3 regions (Stratagene). The generated adenovirus genome can be amplified only within the HEK 293 packaging cell line, complementing the essential regions. Purification of recombinant adenovirus was performed using a cesium chloride gradient, and the adenoviral stocks were quantified using an Adeno-X™ Rapid Titer Kit (Clontech, Saint-Germain-en-Laye, France).

### 2.4. Flow Cytometry

Organs were minced and intrahepatic lymphocytes (IHLs) were separated using a 40%/70% Percoll (GE Healthcare, München, Germany) gradient. Remaining red blood cells were lysed, and lymphocytes were subsequently stained with appropriate combinations of anti-CD3, anti-CD4, anti-CD8, anti-CD62L and anti-CD44. All acquisitions were performed with an LSRII SORP interfaced with FACSDiva software v 8.0.3 (BD Biosciences, Heidelberg, Germany).

### 2.5. Histology

Murine liver was fixed in formalin and embedded. Paraffin-embedded sections (5 µm) were prepared for hematoxylin and eosin (HE) staining. After staining, the sections were examined in a blinded manner by a pathologist using the approved modified hepatitis activity index (mHAI) which also includes portal inflammation.

### 2.6. Protein Detection in the Serum

Proteins were measured using the Olink^®^ MOUSE EXPLORATORY panel* (Olink Proteomics AB, Uppsala, Sweden), according to the manufacturer’s instructions as described before [14]. The proximity extension assay (PEA) technology used for the Olink protocol has been well described [15], and enables 92 analytes to be analyzed simultaneously.

### 2.7. Statistics

Unpaired Student 2-tailed *t* tests and heat maps analysis were performed using GraphPad Prism version 7.00 for Mac (GraphPad Software, La Jolla, CA, USA). Alternatively, for multiple variables, the unpaired Student’s 2-tailed *t*-test with an implemented Benjamini–Hochberg multiplicity correction was performed using Qlucore Omics Explorer software 3.5 (Qlucore, Lund, Sweden). Heat maps represent the multiple protein expression profiles (*p* < 0.05; *q* < 0.05). * significant difference with *p* ≤ 0.05; ** very significant difference, *p* ≤ 0.01; *p* > 0.05 was considered to be not significant (ns).

## 3. Results

### 3.1. The Spleen Has a Protective Role in the Induction Phase of Autoimmune Hepatitis

Experimental murine AIH is induced by intravenous Ad-FTCD application [3,16]. Analyses of animals were performed twelve weeks after adenoviral induction of emAIH. Recently, we have shown that the spleen is not the site where emAIH is induced [13]. Furthermore, we speculated as to whether the spleen might have a protective function for autoimmune diseases, especially emAIH. 

**Figure 1:**
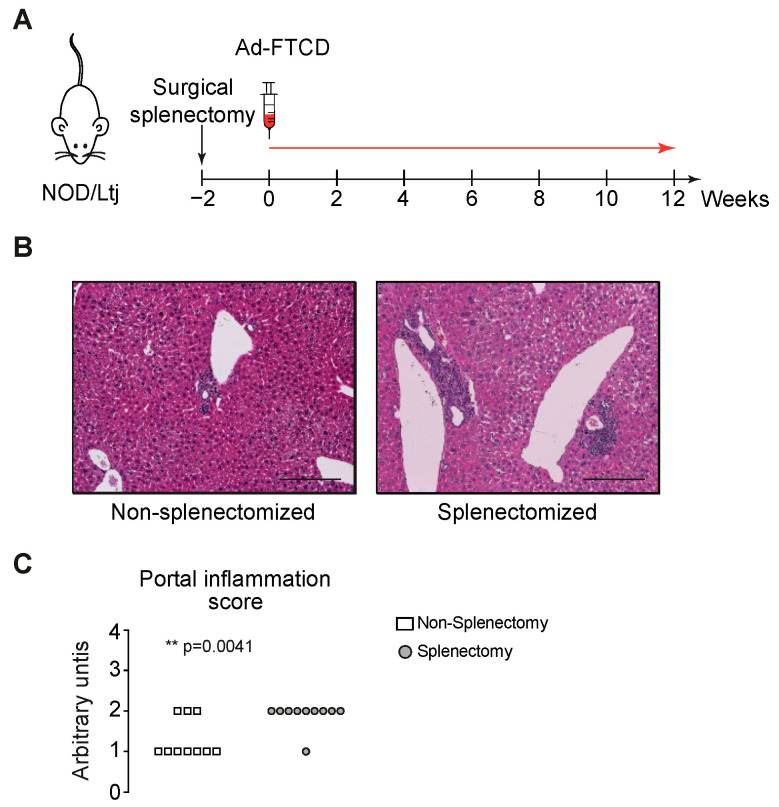
Increased portal inflammation in emAIH after splenectomy. (**A**) emAIH Model with splenectomy two weeks before induction in NOD/Ltj mice. (**B**) HE staining was performed with liver sections of splenectomized mice harvested twelve weeks after adenoviral emAIH induction. Scale bar represents 100 µm. (**C**) Liver sections were analyzed for portal inflammation as part of the approved blinded modified hepatitis activity index (mHAI).

Therefore, we splenectomized mice and induced emAIH in these and non-splenectomized controls (Figure 1A). Splenectomy without emAIH induction had no effect on hepatic histology, transaminases, or lymphocyte compartments (data not shown). Both groups showed inflammation twelve weeks after emAIH induction (Figure 1B) [13]. As hypothesized, the splenectomized animals showed more portal inflammation compared to animals with a spleen (Figure 1C).

### 3.2. Increased Portal Inflammation Correlates with Amplified IL-17

**Figure 2:**
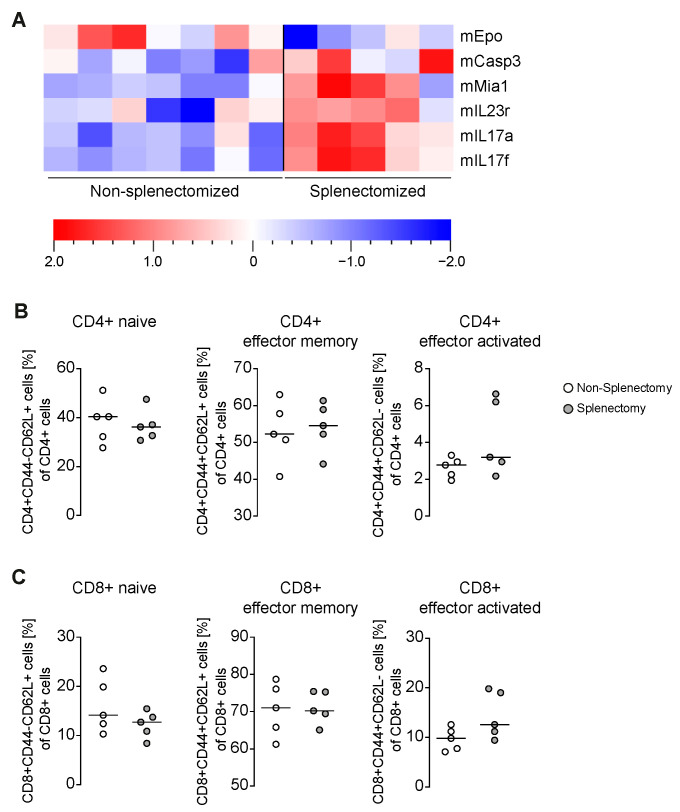
Increased IL-17, IL-23R and casp3 with a lack of Epo in splenectomized mice with emAIH. (**A**) Serum analyses of splenectomized and non-splenetomized NOD/Ltj for 92 proteins by Olink technology. Shown heat-map takes into account the multiplicity correction after calculating the *p* (<0.05) and the q values (<0.05) for all 92 proteins. (**B**) Flow cytometry of intrahepatic CD3^+^CD4^+^ T cells and (**C**) CD3^+^CD8^+^. Shown graphs display mean ± SD; they were all not significant.

We have already shown that the mRNA of inflammatory cytokines, TH1-, TH2-, Th17-, Treg- and fibrosis markers was not differentially regulated [13]. Nonetheless, we analyzed the sera of all mice and quantified 92 different proteins within them. Six of the proteins were differentially regulated (Figure 2A). We observed less Epo in splenectomized emAIH bearing animals, but more melanoma-derived growth regulatory protein (Mia), casp3, IL-23R, IL-17a and IL-17f. Because T cells could be identified as definitive drivers of emAIH [3,5], we analyzed the T cell compartments more in detail. We already published that neither the proportion nor the absolute number of CD4^+^ and CD8^+^ T cells changed [13]. Therefore, we looked more in detail in CD4^+^ (Figure 2B) and CD8^+^ T cells (Figure 2C). Surprisingly, neither the naive, activated nor memory CD4^+^ or CD8^+^ T cells differ in mice splenectomized before emAIH induction, although these have more portal inflammation and more IL-17. 

## 4. Discussion

In this study, we were able to show that the spleen in the induction phase of emAIH tends to favor the course of disease. As main factors associated with the strong portal inflammation in the liver, we could demonstrate a lack of Epo and a very strong increase in casp3, Il-17 and Il-23R. 

As shown, the mHAI of autoimmune hepatitis is even more severe in splenectomized mice [13]. This is due to the increase in portal inflammation (Figure 1C). Whether the strong increase in casp3, Il-17 and IL-23R, as well as the lack of Epo, is a cause or a consequence, is the chicken or the egg causality dilemma. The type I cytokine Epo acts in its best characterized role as a key regulator of erythropoiesis [17,18]. Furthermore, Epo inhibits the apoptosis of erythroid progenitor cells, and is tissue-protective [18,19]. Likewise, the cause for the lack of Epo could be due to the lack of its splenic production or the suspected lack of its hepatic release. This could not be answered in this set-up and would require further studies. 

The pro-apoptotic protein casp3 in AIH is a predictor for the severity of AIH [20]. In various liver diseases, a decrease of casp3 is an important marker for therapy response or the inhibition of casp3 is even beneficial to the course of the disease [21,22,23]. The increased severity of hepatitis can be attributed to a lack of the anti-apoptotic Epo and a surplus of the pro-apoptotic casp3. However, this does not answer the question of causality. 

The fact that AIH is a T-cell driven disease is generally undisputed and could also be shown for different AIH models [3,4,5,6,7,8,24]. Nevertheless, we measured increased IL-17 and IL-23R levels without a serious effect on the T cells (Figure 2B,C), although portal inflammation was increased. This could be a gap in the methodology. Even in the original description of the emAIH model, no differences in the cell population could be detected by flow cytometry. We had to use the ELISPOT technology to find out that the autoreactive cells represented a frequency too low for flow cytometry [3]. The same problem is known from other autoimmune diseases such as type 1 diabetes [25,26]. It is well described by Zhao et al. and others that Th17 cells are the major source of IL-17 in the liver [27,28].

In summary, we could demonstrate that the spleen possesses an immune regulatory effect in the initial phase of emAIH. We could also show that the increase in the mHAI was driven by portal inflammation that might be caused by a lack of anti-apoptotic Epo and increased pro-apoptotic casp3. Finally, we were able to show a large excess of IL-17 and IL-23R, which should have an effect on the T-cell response. The results have important implications for our understanding of the etiology of AIH and possible treatment options that are related to the anti-apoptotic or anti-IL-17 strategies. 

## 5. Conclusions

Induction of experimental murine autoimmune hepatitis occurred in the liver, while the spleen had an immune regulatory function. In this autoimmune hepatitis model, splenectomy prior disease induction increased the amount of IL-17, IL-23R and caspase-3 in the sera of mice with experimental murine autoimmune hepatitis.

## Data Availability

The data presented in this study are available on request from the corresponding author.

## Abbreviations

Ad	adenovirus
AIH	autoimmune hepatitis
emAIH	experimental murine autoimmune hepatitis
Epo	erythropoietin
FTCD	formiminotransferase cyclodeaminase
mHAI	modified hepatitis activity index

## References

[1] Manns M.P., Czaja A.J., Gorham J.D., Krawitt E.L., Mieli-Vergani G., Vergani D., Vierling J.M., American Association for the Study of Liver (2010). Diagnosis and management of autoimmune hepatitis. Hepatology.

[2] European Association for the Study of the Liver (2015). EASL Clinical Practice Guidelines: Autoimmune hepatitis. J. Hepatol..

[3] Hardtke-Wolenski M., Fischer K., Noyan F., Schlue J., Falk C.S., Stahlhut M., Woller N., Kuehnel F., Taubert R., Manns M.P. (2013). Genetic predisposition and environmental danger signals initiate chronic autoimmune hepatitis driven by CD4(+) T cells. Hepatology.

[4] Hardtke-Wolenski M., Taubert R., Noyan F., Sievers M., Dywicki J., Schlue J., Falk C.S., Lundgren B.A., Scott H.S., Pich A. (2015). Autoimmune hepatitis in a murine autoimmune polyendocrine syndrome type 1 model is directed against multiple autoantigens. Hepatology.

[5] Dywicki J., Noyan F., Misslitz A.C., Hapke M., Galla M., Schlue J., Liblau R.S., Taubert R., Manns M.P., Jaeckel E. (2018). Hepatic T Cell Tolerance Induction in An Inflammatory Environment. Dig. Dis..

[6] Lapierre P., Djilali-Saiah I., Vitozzi S., Alvarez F. (2004). A murine model of type 2 autoimmune hepatitis: Xenoimmunization with human antigens. Hepatology.

[7] Holdener M., Hintermann E., Bayer M., Rhode A., Rodrigo E., Hintereder G., Johnson E.F., Gonzalez F.J., Pfeilschifter J., Manns M.P. (2008). Breaking tolerance to the natural human liver autoantigen cytochrome P450 2D6 by virus infection. J. Exp. Med..

[8] Lohse A.W., Obermayer-Straub P., Gerken G., Brunner S., Altes U., Dienes H.P., Manns M.P., Buschenfelde K.H.M.Z. (1999). Development of cytochrome P450 2D6-specific LKM-autoantibodies following liver transplantation for Wilson’s disease—Possible association with a steroid-resistant transplant rejection episode. J. Hepatol..

[9] Backer R., Schwandt T., Greuter M., Oosting M., Jungerkes F., Tuting T., Boon L., O’Toole T., Kraal G., Limmer A. (2010). Effective collaboration between marginal metallophilic macrophages and CD8+ dendritic cells in the generation of cytotoxic T cells. Proc. Natl. Acad. Sci. USA.

[10] Aoki N., Kido M., Iwamoto S., Nishiura H., Maruoka R., Tanaka J., Watanabe T., Tanaka Y., Okazaki T., Chiba T. (2011). Dysregulated generation of follicular helper T cells in the spleen triggers fatal autoimmune hepatitis in mice. Gastroenterology.

[11] Bowen D.G., Zen M., Holz L., Davis T., McCaughan G.W., Bertolino P. (2004). The site of primary T cell activation is a determinant of the balance between intrahepatic tolerance and immunity. J. Clin. Investig..

[12] Derkow K., Loddenkemper C., Mintern J., Kruse N., Klugewitz K., Berg T., Wiedenmann B., Ploegh H.L., Schott E. (2007). Differential priming of CD8 and CD4 T-cells in animal models of autoimmune hepatitis and cholangitis. Hepatology.

[13] Dywicki J., Buitrago-Molina L.E., Pietrek J., Lieber M., Broering R., Khera T., Schlue J., Manns M.P., Wedemeyer H., Jaeckel E. (2020). Autoimmune hepatitis induction can occur in the liver. Liver Int..

[14] Romermann D., Ansari N., Schultz-Moreira A.R., Michael A., Marhenke S., Hardtke-Wolenski M., Longerich T., Manns M.P., Wedemeyer H., Vogel A. (2020). Absence of Atg7 in the liver disturbed hepatic regeneration after liver injury. Liver Int..

[15] Assarsson E., Lundberg M., Holmquist G., Bjorkesten J., Thorsen S.B., Ekman D., Eriksson A., Dickens E.R., Ohlsson S., Edfeldt G. (2014). Homogenous 96-plex PEA immunoassay exhibiting high sensitivity, specificity, and excellent scalability. PLoS ONE.

[16] Hardtke-Wolenski M., Dywicki J., Fischer K., Hapke M., Sievers M., Schlue J., Anderson M.S., Taubert R., Noyan F., Manns M.P. (2017). The influence of genetic predisposition and autoimmune hepatitis inducing antigens in disease development. J. Autoimmun..

[17] Paulson R.F. (2019). Epo receptor marks the spot. Blood.

[18] Nairz M., Sonnweber T., Schroll A., Theurl I., Weiss G. (2012). The pleiotropic effects of erythropoietin in infection and inflammation. Microbes Infect..

[19] Ghezzi P., Brines M. (2004). Erythropoietin as an antiapoptotic, tissue-protective cytokine. Cell Death Differ..

[20] Czaja A.J. (2014). Targeting apoptosis in autoimmune hepatitis. Dig. Dis. Sci..

[21] Bantel H., Lugering A., Poremba C., Lugering N., Held J., Domschke W., Schulze-Osthoff K. (2001). Caspase activation correlates with the degree of inflammatory liver injury in chronic hepatitis C virus infection. Hepatology.

[22] Wang M.C., Wandrer F., Schlue J., Voigtlander T., Lankisch T.O., Manns M.P., Bantel H., von Hahn T. (2018). Transjugular diagnostics in acute liver failure including measurements of hepatocentral venous biomarker gradients. Hepatol. Res..

[23] Thapaliya S., Wree A., Povero D., Inzaugarat M.E., Berk M., Dixon L., Papouchado B.G., Feldstein A.E. (2014). Caspase 3 inactivation protects against hepatic cell death and ameliorates fibrogenesis in a diet-induced NASH model. Dig. Dis. Sci..

[24] Ma Y., Bogdanos D.P., Hussain M.J., Underhill J., Bansal S., Longhi M.S., Cheeseman P., Mieli-Vergani G., Vergani D. (2006). Polyclonal T-cell responses to cytochrome P450IID6 are associated with disease activity in autoimmune hepatitis type 2. Gastroenterology.

[25] Pugliese A. (2017). Autoreactive T cells in type 1 diabetes. J. Clin. Investig..

[26] Martinuzzi E., Novelli G., Scotto M., Blancou P., Bach J.M., Chaillous L., Bruno G., Chatenoud L., van Endert P., Mallone R. (2008). The frequency and immunodominance of islet-specific CD8+ T-cell responses change after type 1 diabetes diagnosis and treatment. Diabetes.

[27] Zhao L., Tang Y., You Z., Wang Q., Liang S., Han X., Qiu D., Wei J., Liu Y., Shen L. (2011). Interleukin-17 contributes to the pathogenesis of autoimmune hepatitis through inducing hepatic interleukin-6 expression. PLoS ONE.

[28] Abe M., Hiasa Y., Onji M. (2013). T helper 17 cells in autoimmune liver diseases. Clin. Dev. Immunol..

